# Fatty Liver Index Associates with Relative Sarcopenia and GH/ IGF- 1 Status in Obese Subjects

**DOI:** 10.1371/journal.pone.0145811

**Published:** 2016-01-07

**Authors:** Eleonora Poggiogalle, Carla Lubrano, Lucio Gnessi, Stefania Mariani, Andrea Lenzi, Lorenzo Maria Donini

**Affiliations:** Department of Experimental Medicine- Medical Pathophysiology, Food Science and Endocrinology Section, “Sapienza” University of Rome, Rome, Italy; Dasman Diabetes Institute, KUWAIT

## Abstract

**Introduction:**

Recently the association between hepatic steatosis and sarcopenia has been described. GH/IGF-1 axis has been postulated to play a role in linking fatty liver and low muscle mass. The aim of our study was to explore the association between fatty liver index, sarcopenic obesity, insulin sensitivity, and GH/IGF-1 status.

**Methods:**

427 subjects [age: 45.65±13.94 years, BMI: 36.92±6.43 kg/m^2^] were enrolled. Participants were divided into three groups: fatty liver index (FLI) <20, 20≥FLI<60, and FLI≥60. Body composition was assessed by DXA. The truncal fat mass (TrFM) to appendicular skeletal muscle (ASM) ratio was used as an indicator of sarcopenic obesity. ISI-Matsuda index was used.

**Results:**

BMI, fat mass, and the TrFM/ASM ratio were higher in subjects with FLI≥60. GH, IGF-1 and ISI-Matsuda were lower in the high FLI group (all p<0.05). A significantly positive correlation between FLI and TrFM/ ASM ratio (r = 0.221, p<0.001) was found, whereas FLI levels were negatively correlated with ISI- Matsuda (r = -0.335, p<0.001), GH (r = -0.200, p = 0.006), and IGF- 1 levels (r = -0.157, p = 0.028). Stepwise linear regression analysis showed that GH levels were significantly negatively correlated with FLI, while the TrFM/ ASM ratio was positively associated with FLI, after adjustment for age, BMI, total fat mass, truncal fat mass, fat- free mass, and ISI- Matsuda.

**Conclusions:**

Impairment of GH/IGF-1 axis seems to be associated to the risk of the development of sarcopenic obesity and ectopic fat deposition in the liver. Metabolic and hormonal derangements as determinants of ectopic fat deposition and body composition deserve to be evaluated in obese subjects.

## Introduction

In the last decades, a growing body of evidence dealt with two important issues in the Western world: sarcopenia and obesity, two faces of the challenge of healthy aging [1–3]. Moreover, interest is mounting towards sarcopenic obesity, in which the two abovementioned conditions coexist in a phenotype characterized by high adiposity and low lean body mass [3, 4].

Obesity is known to be strictly linked to fatty liver [5]. Recently, several studies reported an association between low skeletal muscle mass and hepatic steatosis [6, 7]. Insulin resistance has been demonstrated to occur in case of excess fat as well as in sarcopenia, given that skeletal muscle is one of the major target tissues of insulin action [8, 9]. Anyway, the impairment of insulin sensitivity may not be the only mediator of the development of fatty liver, and the role of other potential contributors needs to be better clarified.

Growth hormone (GH) and insulin- like growth factor 1 (IGF- 1) exert significant effects on body composition, and lipid and glucose homeostasis [10]. A wealth of studies reported that in obese subjects GH secretion is blunted [10–12], and GH and IGF- 1 have been proven to be involved in fatty infiltration of the liver [13, 14]. Likewise, ectopic fat deposition seems to be linked to reduced GH levels [10, 15], as well as impairment in GH production occurring with aging is one of the causes of the changes in body compartments leading to both excess adiposity and sarcopenia [16].

More recently, GH has been postulated to play a role in the connection between nonalcoholic fatty liver disease (NAFLD) and decline in muscle mass [17]. The aim of our study was to investigate the association between fatty liver index, the phenotype of sarcopenic obesity, and GH/ IGF- 1 status.

## Materials and Methods

Study participants were recruited among subjects referring to the High Specialization Center for the Care of Obesity at the Department of Experimental Medicine, “Sapienza” University of Rome, Italy, from June 2011 to October 2014.

The inclusion criteria were: age between 18 and 65 years, diagnosis of obesity based on fat mass > 25% in men and >35% in women [18], ethnicity: Caucasian Italian subjects. As exclusion criteria, we considered: malignant disease during the last 5 years, inflammatory or autoimmune diseases, corticosteroids for systemic use, any medication or any clinical condition potentially affecting body weight or body composition, syndromic obesity, participation in a reducing-weight program in the last three months, renal failure, heart failure, any type of diabetes, history of viral or autoimmune liver disease or any other chronic liver disease, excessive alcohol intake (> 140g/ week for men and 70g/ week for women).

The study protocol was approved by the Ethical Committee of the "Sapienza” University of Rome, Italy, and the written informed consent was obtained from all the participants.

All subjects underwent a multidimensional evaluation, as follows.

Complete physical examination and anthropometric measurements. Body weight, height, waist circumference were measured following the procedures described in the “Anthropometric standardization reference manual” [19]. An inter- assessor alignment training session preceded the measurements. The same tools were used in all subjects: a SECA scale 86 (200 kg, to the nearest 0.1 kg), a flexible metallic tape (200 cm, to the nearest 0.1 cm), a telescopic stadiometer (200 cm; to the nearest 0.1 cm). Body mass index (BMI) was calculated as body weight (kg) divided by height squared (m^2^).

Body composition analysis. Fat mass and fat- free mass were assessed by dual-energy-X-ray absorptiometry (DXA) (Hologic 4500 RDR), with coefficient of variation of < 1.5% for fat mass and fat- free mass. Segmental regions were also measured. Truncal fat mass (TrFM) was assessed. Appendicular skeletal muscle mass (ASM) was evaluated by DXA and calculated as the sum of lean soft tissue masses of arms and legs [20].

The ratio between truncal fat mass and appendicular skeletal muscle mass (TrFM/ ASM ratio) was calculated and used as an index of sarcopenic obesity [21].

Biochemistry. Blood samples were collected after an overnight fast. The following biochemical parameters were assayed: fasting glucose and insulin, alanine aminotransferase (ALT), aspartate aminotransferase (AST), gamma- glutamyl transferase (GGT), triglycerides, GH and IGF- 1, using commercial kits.

Glucose metabolism and insulin sensitivity. All participants underwent an oral glucose tolerance test (OGTT) for both glucose and insulin response. Insulin sensitivity was assessed as the insulin sensitivity index (ISI) calculated using the OGTT values as proposed by Matsuda and DeFronzo [22].

The fatty liver index (FLI) was calculated as follows [23]:

$$\begin{array}{l} (\text{FLI}\;=\;(e\;0.953*loge\;\left(triglycerides\right)\;+\;0.139*BMI\;+\;0.718\\ {}*loge\;\left(ggt\right)\;+\;0.053*waist\;circumference\;-\;15.745)\;/\;(1\\ +\;e0.953*loge\;\left(triglycerides\right)\;+\;0.139*BMI\;+\;0.718\\ {}*loge\;\left(ggt\right)\;+\;0.053*waist\;circumference\;-\;15.745)\;*\;100) \end{array}$$

Participants were divided into three groups, according to FLI values: FLI < 20, 20≥ FLI < 60, and FLI ≥ 60 [24].

### Statistical analysis

After verification of normal data distribution, T- test was used to compare continuous variables, χ^2^ was applied to compare categorical variables. A univariate analysis was performed in order to investigate the relationship among the outcome variable (FLI) and the independent variables: GH, IGF- 1, TrFM/ ASM ratio for body composition, ISI- Matsuda for insulin sensitivity (Pearson’s correlation coefficient). The predictors of the outcome variable, significantly correlated at the univariate analysis, were included in a multiple linear regression model to verify the association among FLI and the independent variables.

The regression model was elaborated using a stepwise (cutoff for entry: 0.05, for removal: 0,10) procedure. The efficacy of the regression model was analyzed according to the value of the determination coefficient R^2^ (comparing the explained variance of the model’s predictions with the total variance of the data) and the R^2^ adjusted (considering a correction for inclusion of variables). The standard error of the estimate (SEE), representing a measure of the accuracy of predictions (standard deviation of the differences between the actual values of the dependent variable and the predicted values), was calculated. A p value < 0.05 was considered statistically significant.

Data were entered a Microsoft Excel database and analyzed using the statistical software SPSS for Windows 10.0 (SPSS Inc. 1989–1999).

## Results

427 subjects (81 men and 346 women, mean age: 48.17 ± 14.84 and 45.06 ± 13.68 years, mean BMI: 37.31 ± 6.40 and 36.83 ± 6.45 kg/m^2^, respectively) were enrolled. Only one man and 6 women presented a FLI value ≤ 20, thus we excluded them from the statistical analysis. The intermediate and the high FLI groups were compared. Characteristics of study participants are described in Table 1.

**Table 1:** Demographic, anthropometric and metabolic characteristics of study participants (N = 420).

	FLI 20–60 (N = 61)	FLI > 60 (N = 359)	p
**Age (years)**	42.69±14.48	46.20±13.80	0.058
**BMI (Kg/m**^**2**^**)**	29.59±2.22	38.29±6.02	<0.001
**Waist circumference (cm)**			
** M**	97.50±4.09	122.11±11.96	0.001
** F**	98.07±7.02	118.61±12.01	<0.001
**Fat mass (kg)**			
** M**	22.40±0.69	36.30±7.63	0.002
** F**	31.08±4.27	43.07±9.10	<0.001
**Fat mass (%)**			
** M**	25.83±0.84	31.92±4.17	0.014
** F**	40.10±3.94	42.78±4.47	<0.001
**Fat- free mass (kg)**			
** M**	59.52±4.10	72.82±10.09	0.014
** F**	44.36±5.70	53.53±8.13	<0.01
**Fat- free mass (%)**			
** M**	74.17±0.84	68.03±4.17	0.013
** F**	59.91±3.94	57.22±4.47	<0.001
**Truncal fat mass (kg)**			
** M**	11.11±1.12	19.14±4.34	0.002
** F**	13.81±2.21	20.63±4.69	<0.001
**Truncal fat mass (%)**			
** M**	27.10±1.87	34.23±4.86	0.014
** F**	38.08±4.76	42.72±5.54	<0.001
**ASM (kg)**			
** M**	26.23±2.41	32.22±4.78	0.035
** F**	18.88±2.88	23.04±3.95	<0.001
**TrFM/ASM ratio**			
** M**	0.43±0.04	0.61±0.14	0.003
** F**	0.75±0.15	0.91±0.22	<0.001
**GH (ug/mL)**	2.08±0.79	0.92±0.74	0.008
**IGF-1 (ng/mL)**	216.80±85.29	176.08±92.63	0.031
**ISI- Matsuda**	5.03±3.14	3.03±2.27	<0.001
**HbA1c (%)**	5.36±0.44	5.97±1.12	<0.001
**Triglycerides (mg/dL)**	98.49±65.16	130.36±75.65	<0.001
**AST (U/L)**	17.51±4.99	22.78±12.70	<0.001
**ALT (U/L)**	19.39±8.11	30.43±21.92	<0.001
**GGT (U/L)**	16.74±10.07	28.93±23.56	<0.001

**Legend**: FLI = fatty liver index; BMI = body mass index; ASM = appendicular skeletal muscle; TrFM = truncal fat mass; GH = growth hormone; IGF- 1 = insulin- like growth factor 1; ISI = insulin sensitivity index; HbA1c = glycosylated haemoglobin; ALT = alanine aminotransferase, AST = aspartate aminotransferase, GGT = gamma- glutamyl transferase.

Age was not significantly different between the groups. BMI, waist circumference, absolute body fat mass, body fat mass percentage, truncal fat mass, absolute fat- free mass, fat- free mass percentage, appendicular skeletal muscle mass and the TrFM/ ASM ratio were significantly higher in subjects with FLI ≥ 60, when compared with individuals in the intermediate FLI group, regardless of gender. GH and IGF- 1 were significantly lower in subjects with FLI values ≥ 60. Concerning insulin sensitivity, ISI- Matsuda was significantly reduced in the high FLI group (all p values < 0.05).

A significantly positive correlation between FLI and TrFM/ ASM ratio (r = 0.221, p<0.001) was observed (Table 2). On the contrary, FLI levels were negatively correlated with ISI- Matsuda (r = -0.335, p<0.001), GH (r = -0.200, p = 0.006), and IGF- 1 levels (r = -0.157, p = 0.028).

**Table 2:** Univariate correlation analysis among FLI and explanatory variables.

Variables	r	p
**Age (years)**	0.131	0.007
**BMI (kg/m**^**2**^**)**	0.691	<0.001
**FM (kg)**	0.586	<0.001
**Truncal FM (kg)**	0.662	<0.001
**FFM (kg)**	0.540	<0.001
**TrFM/ ASM ratio**	0.221	<0.001
**GH (ug/mL)**	-0.200	0.006
**IGF-1 (ng/mL)**	-0.157	0.028
**ISI- Matsuda**	-0.335	<0.001

**Legend:** FLI = fatty liver index; TrFM/ ASM = truncal fat mass/ appendicular skeletal muscle; GH = growth hormone; IGF- 1 = insulin- like growth factor 1; ISI = insulin sensitivity index

Stepwise linear regression analysis showed that GH levels were significantly negatively correlated with FLI, while the TrFM/ ASM ratio was positively associated with FLI, after adjustment for age, BMI, total fat mass, truncal fat mass, fat- free mass, and ISI- Matsuda (Table 3).

**Table 3:** Stepwise linear regression analysis using FLI as dependent variable.

Model	Unstandardized Coefficients^§^		Standardized coefficients	t	p
	B	SE	Beta		
**GH (ug/mL)**	-1.267	0.505	-0.133	-2.508	0.013
**TrFM/ ASM ratio**	25.095	5.953	0.271	4.215	<0.001
**(Costant)**	-31.850	9.823	n. a.	-3.242	0.002
**R**^**2**^ = 0.734; **R**^**2**^ **adj**. = 0.720; **SEE** = 11.13					

^§^ After adjustment for age, BMI, total FM, FFM, truncal FM, and ISI- Matsuda

**Legend:** FLI = fatty liver index; GH = growth hormone; TrFM/ ASM = truncal fat mass/ appendicular skeletal muscle; SE = standard error; SEE = standard error of the estimate; BMI = body mass index; FM = fat mass; FFM; = fat- free mass, ISI = insulin sensitivity index; n.a. = not available.

## Discussion

Separate associations between GH and IGF- 1 and hepatic steatosis, as well as connections between GH pathway and metabolic response in adipose tissue and skeletal muscle are well established. Only few studies described the association between sarcopenia and NAFLD [6,7]. However, the complex interplay between body composition, fatty infiltration of the liver, GH status and insulin sensitivity has not been thoroughly investigated to date.

In the present study, we found a significant positive association between indices of fatty liver and of sarcopenic obesity, and an inverse association between fatty liver index, sarcopenic obesity phenotype, and GH status.

Consistent with previous studies, showing reduced levels of GH and IGF- 1 in case of NAFLD [13,14] and obesity [10], our study population showed decreased circulating amounts of GH and IGF- 1 in obese subjects with FLI values higher than 60. GH and IGF- 1 correlated negatively and TrFM/ ASM ratio correlated positively with FLI in the univariate analysis. Body size and body composition, especially fat mass, are known to influence individual components of FLI (e.g. BMI and waist circumference) as well as GH status; on the other hand, insulin resistance is notably linked to NAFLD. In the final model of multivariate regression, the positive correlation of TrFM/ ASM ratio with FLI, and the negative correlation of GH with FLI as dependent variable were maintained after adjusting the statistical model for other variables potentially affecting this association, such as age, BMI, body composition (total fat mass, truncal fat mass, fat- free mass) as well as ISI- Matsuda. Even though IGF- 1 is a mediator of GH effects, it was excluded in the model because of collinearity [10, 16].

Our results seem to be in line with findings from Bredella et al. [25], showing an inverse association between GH and intrahepatic lipid content in obese premenopausal women independently of visceral fat and age. Similarly, in two other studies including Caucasian subjects NAFLD detected by ultrasound was negatively associated with IGF- 1 levels after controlling for BMI [26, 27], on the contrary, the strength of this relationship was attenuated when adjusting for adiposity in the study by Runchey et al.[28], involving normal-weight- to- obese subjects of mixed ethnicity.

Consistent with data from the Korean Sarcopenic Obesity Study [7], showing a higher risk of NAFLD in individuals with lower muscle mass after adjustment for HOMA- IR, we found a significant inverse association between fatty liver index and the index of sarcopenic obesity TrFM/ASM ratio after controlling for insulin resistance. In particular, we used the ISI- Matsuda that mirrors more exhaustively the impairment of insulin sensitivity [29]. Accordingly, various studies showed that insulin resistance is involved in the development of NAFLD and is related to sarcopenia [9, 30, 31].

In line with previous data showing a poor control of glucose metabolism in subjects with FLI ≥ 60 [6], our results suggest that in obese individuals in the high FLI group, the concomitant presence of sarcopenia may worsen the negative metabolic effects of obesity on glucose homeostasis.

As reported just by two other studies [6,7], we found a positive relationship between fatty liver index and low relative skeletal muscle mass.

In our patients in the high FLI group, the absolute fat- free mass was increased. This observation may be explained by the fact that in obese subjects, in an adaptive mechanism counteracting the increased load of fat mass, approximately 25% of the body mass consists of fat- free tissues [32]. This homeostatic response may not be sufficient in functional and metabolic terms in obese individuals leading to sarcopenic obesity [21]. Moreover, although not decreased, absolute fat- free mass in our obese subjects was likely infiltrated by fat, as an inverse association between GH status and thigh intramuscular fat was described [15]. Besides, according to others [10, 33], the reduced GH and IGF- 1 levels seen in our population of obese patients, may be detrimental both to liver and skeletal muscle, contributing to the ectopic fat storage. On the basis of the above mentioned considerations, our findings are in line with the hypothesis raised by Guichelaar and Charlton [17] suggesting GH status as a potential bridge between fatty liver and sarcopenia.

A universally accepted definition of sarcopenic obesity is lacking to date. The majority of existing definitions of sarcopenic obesity relies on criteria considering sarcopenia and obesity separately [4, 34]; most of these indices have been validated as opposed to functional impairment, while just few recent studies focused on metabolic aspects [4, 34–37]. In the present study we used the TrFM/ASM ratio to describe the sarcopenic obesity phenotype. This index, that includes both fat mass and fat- free mass in a unique estimate, better captures the metabolic features due to the disproportion in body compartments occurring in sarcopenic obesity [21]. In addition, we defined obesity on the basis of the measured fat mass, a better criterion for the diagnosis of obesity compared to an indirect index like BMI [18, 30].

Some limitations to our study need to be considered. Although the FLI has been validated against hepatic ultrasonography and magnetic resonance spectroscopy [23, 38], it is a surrogate marker of liver steatosis. Moreover, just a minority of obese subjects in our study population had FLI values below 20. The stratification of FLI based on cut-off values seems not to be completely appropriate for our cohort of obese subjects (mostly females), whose mean BMI was higher than BMI in the validation study of FLI (largely males) [23], as well as in the study by Gastaldelli and coll., using the same cut-off values than in our study [24]. In a recent study by Cuthbertson and al. [38], FLI was linearly related to log- transformed liver fat assessed by magnetic resonance spectroscopy, and was confirmed to be useful to identify subjects with hepatic steatosis, even though it cannot be used to predict exactly the liver fat content. For these reasons, we used FLI as a continuous variable in the regression analysis. Finally, our analysis does not prove a causal relationship among GH status, sarcopenic obesity and fatty liver.

The lack of a separate analysis of sarcopenic and nonsarcopenic obese subjects taking into account sex hormones may represent another limitation to our study. Nevertheless, our study population was composed mainly by adult subjects; evidence on changes in sex hormones and body composition was provided especially by studies involving subjects in the menopausal state and in the geriatric age (typically >65 years old). However, in the European Male Ageing Study, no consistent association was observed between testosterone levels and change in muscle mass, gait speed, or grip strength in middle-aged and elderly men [39].

In conclusion, our findings encompass previous evidence into a more comprehensive point of view that relates metabolic and hormonal derangements as determinants of ectopic fat deposition and body composition.

Obese subjects should be evaluated for metabolic features linked to fatty infiltration of liver and muscle in particular. In addition, specific treatment strategies for the phenotype of sarcopenic obesity should be adopted, to prevent detrimental changes in body composition due to the altered GH/ IGF- 1 status.

## Abbreviations

GH: growth hormone
IGF- 1: insulin- like growth factor 1
NAFLD: nonalcoholic fatty liver disease
BMI: body mass index
DXA: dual- energy- X- ray absorptiometry
TrFM: truncal fat mass
ASM: appendicular skeletal muscle mass
TrFM/ ASM ratio: truncal fat mass and appendicular skeletal muscle mass ratio
ALT: alanine aminotransferase
AST: aspartate aminotransferase
GGT: gamma- glutamyl transferase
OGTT: oral glucose tolerance test
ISI: insulin sensitivity index
FLI: fatty liver index
HOMA- IR: homeostasis model assessment of insulin resistance
